# Multiplex communities and the emergence of international conflict

**DOI:** 10.1371/journal.pone.0223040

**Published:** 2019-10-16

**Authors:** Caleb Pomeroy, Niheer Dasandi, Slava Jankin Mikhaylov

**Affiliations:** 1 Department of Political Science, The Ohio State University, Columbus, Ohio, United States of America; 2 School of Government, University of Birmingham, Birmingham, United Kingdom; 3 Data Science Lab, Hertie School, Berlin, Germany; University of Oxford, UNITED KINGDOM

## Abstract

Advances in community detection reveal new insights into multiplex and multilayer networks. Less work, however, investigates the relationship between these communities and outcomes in social systems. We leverage these advances to shed light on the relationship between the cooperative mesostructure of the international system and the onset of interstate conflict. We detect communities based upon weaker signals of affinity expressed in United Nations votes and speeches, as well as stronger signals observed across multiple layers of bilateral cooperation. Communities of diplomatic affinity display an expected negative relationship with conflict onset. Ties in communities based upon observed cooperation, however, display no effect under a standard model specification and a positive relationship with conflict under an alternative specification. These results align with some extant hypotheses but also point to a paucity in our understanding of the relationship between community structure and behavioral outcomes in networks.

## Introduction

Community structure is a fundamental feature of complex networks. The community detection task consists of the identification of subgraphs where vertices exhibit dense within-group ties relative to out-group ties [1]. These mesostructural patterns shed light on physical, biological, and social networks, with applications ranging from disease surveillance to paper citations [2–9]. Early work on modularity developed a principled assessment of the quality of network divisions [10–12], and the current battery of detection tools permits investigation of multilayer, multiplex, and time-dependent networks, including algorithms that can accommodate signed edges and heterogeneously structured networks [13–18].

For computational social scientists, this methodological expansion permits investigation of theoretical questions that previously posed modeling challenges at the mesostructural level. The enduring debate on the relationship between interconnectedness and conflict in International Relations (IR) is an exemplary case. On the one hand, Jean-Jacques Rousseau believed that “…interdependence breeds not accommodation and harmony, but suspicion and incompatibility” ([19] page 321). More recently, Kenneth Waltz argued that “the fiercest civil wars and the bloodiest international ones have been fought within arenas populated by highly similar people whose affairs had become quite closely knit together” ([20] page 138). On the other hand, Immanuel Kant emphasized “that the growth of interconnectedness demonstrated the existence of the unique human capacity for establishing systems of cooperation…” [21]. More contemporary liberal IR theorists also stress the pacifying effects of interdependence [22, 23].

Empirical investigations of this question typically conceptualize interdependence at the dyad level—such as trade flow ratios between states *v*_*i*_ and *v*_*j*_—and infer relationships to conflict via (generalized) linear models, e.g. [24, 25]. This conceptualization implies, for example, that if three states *v*_*i*_, *v*_*j*_, and *v*_*k*_ enjoy a closed triadic cooperation agreement and *v*_*i*_ reneges on the agreement, the exit of state *v*_*i*_ from the commitment to *v*_*j*_ is independent of the exit of state *v*_*i*_ from the commitment to *v*_*k*_. This introduces statistical issues associated with the use of dyads to study *k*-adic phenomena [26] and misses the fundamental mechanism of theoretic interest, or as Lupu & Traag ([27] page 1012) put it: “…[scholars] have assumed independence in order to study interdependence”. Indeed, it has been suggested that until we “create and test more complex models, we are not likely to make theoretical progress in sorting out this question” ([28] page 56).

We draw upon two recent developments relevant to the question of interconnectedness and conflict. First, in international politics, an emerging literature deploys community detection algorithms to examine the role of trade, democracy, and intergovernmental organization dependencies [27, 29], as well as separate attention to alliances [13] and UN votes [30]. The common intuition underlying each of these studies is that the community structure of the international system is an underdeveloped predictor of behavioral outcomes. Second, recent findings in the broader network cooperation literature suggest that community structure helps to explain the emergence and maintenance of cooperation on graphs [31, 32] and that multilayer and multiplex structure fosters cooperative stability [33, 34]. These findings are important for network analytic approaches to international politics, because in contrast to laboratory settings with well-mixed populations, states are indeed embedded in multiple layers of potentially interdependent relations. The network cooperation literature, however, has less to say about the relationship between multiplex community structure and other behavioral outcomes, such as conflict.

This paper employs advances in multilayer community detection to locate dense clusters of states and then inferentially models these communities against the emergence of conflict in the international system. Previous work finds pacifying effects of community membership in the traditional Kantian-inspired foci of trade, democracy, and intergovernmental organization networks [27, 29]. We innovate through attention to data beyond these networks in order to better define the scope of the beneficial effects of community membership on conflict: does the broader cooperative mesostructure of the international system display similar effects, or are previous findings contingent on Kantian-based networks in particular? We consider weaker signals of expressed affinity in the United Nations (UN), as well as stronger signals of observed bilateral cooperation agreements. For the former, we employ layers of UN votes and speeches. For the latter, we search across network layers of science, military, commodity, fishery, and telecommunication cooperation agreements.

The results suggest the following. First, diplomatic cohesion in UN votes and speeches associates negatively with conflict onset. That is, the presence of an affinity community tie in a given dyad correlates with a decrease in conflict likelihood within that dyad. This result provides an extension to a previous finding based upon UN votes alone through the addition of diplomatic speeches in a multilayer setting [30]. Second, states embedded in cooperation communities appear no more or less likely to engage in conflict under a standard model specification and are more likely to engage in conflict under an alternative specification. This finding contrasts with the often implicit assumption that cooperation community membership reduces the likelihood of conflict amongst members. Furthermore, states who bridge multiple cooperation communities are significantly more likely to experience conflict. These findings lend some support for extant hypotheses but also point to a paucity in current knowledge about the relationship between community structure and behavioral outcomes in social systems.

## Results

### Community detection procedure

We follow recent work in conceptualizing the international system as a multilayer network [29]: a network representation where nodes are connected across layers of different tie sets [35–37]. Whereas a single mode representation is especially useful for the isolation of specific theoretical mechanisms (e.g. a trade tie’s impact on *Y*), we instead aim to capture broader cooperative structure that might exist across layers of the international system. Yet, because innumerable slices of relationships exist in international politics, the resulting communities can quickly become uninterpretable. We therefore focus on two types of multilayer graphs based upon data previously scrutinized by network analysts in IR, namely bilateral cooperation agreements and position affinity expressed in the UN. The former represent stronger signals of observed country-country relations, whereas the latter represent weaker, correlational signals of affinity in expressed preferences.

For strong signal communities, we employ five cooperation topics from the World Treaty Index: science, military, commodities, fisheries, and telecommunications [38, 39]. Previous research finds that network dynamics in part drive bilateral agreement formation and evolution on these topics [40]. These topics represent key areas of coordination [41, 42] and help to avoid redundancy across layers due to their relative orthogonality. For example, state motivations behind fishery agreement formation differ from motivations behind science agreement formation [43]. This topical diversity increases confidence that detected communities represent groups of intensive cooperators across issue areas.

For each year, we take the multilayer graph $G_{t}=\left(V,E\right)=\left\{G_{t_{1}},\ldots ,G_{t_{k}}\right\}$, *i* ∈ {1, …, *k*} where $G_{t_{i}}=\left(V,E\right)$ is a single elementary network layer that corresponds to one of the five distinct topics. Each layer contains an aligned node set V=V with an undirected and unweighted edge *e*_*ij*_ = *e*_*ji*_ = (*v*_*i*_, *v*_*j*_) ∈ *E* between nodes *v*_*i*_ and *v*_*j*_ if there exists a bilateral agreement between these two countries in layer $G_{t_{i}}$. We use a moving window such that an edge is present if a bilateral agreement was initiated within the past ten years, and we assume that the edge dissipates outside of this window. This provides a sequence of yearly multilayer graphs $S_{G_{t}}=\left\{G_{1},\ldots ,G_{t}\right\}$.

For weak signal communities, we employ UN votes and a recently released dataset of speeches delivered during the annual UN General Debate [44]. UN votes represent a key source of information about the expressed preferences of states [45–48]. Furthermore, previous network research examines UN voting communities in detail [49], including the relationship between community membership and conflict [30]. In contrast to previous community detection work, however, we employ country ideal points rather than raw UN votes. Noting methodological challenges associated with UN votes, Bailey et al [48] propose the use of unidimensional ideal points estimated from a dynamic ordinal spatial model. Thus, ideal points derive from a more theoretically-informed model of vote choice given a state’s preferences. For each year, we calculate the Euclidean distance between each country pair’s ideal points, converting each distance to a similarity score in order to construct a *V* × *V* similarity matrix.

We utilize speeches as the second graph layer in order to align with recent political science research that turns to text data in order to more accurately capture the expressed positions of political actors, e.g [50–53]. UN votes often display high cohesion, with states casting votes along regional bloc lines, for ceremonial purposes, or because specific agenda items arise beyond the state’s control [44, 47]. State speeches, on the other hand, provide delegations with greater flexibility to express positions. For example, in 1974 Greece and Turkey voted the most similarly amongst NATO members in the UN General Assembly (with ideal points of 0.68 and 0.42, respectively). Yet, that same year the two country’s air forces engaged in a dogfight which led to the death of a Turkish pilot during tensions that arose from Turkey’s invasion of Cyprus. In contrast to their votes, their UN General Debate speeches revealed these tensions, with each blaming the other for the crisis. The Supplementary Information (SI) describes this example and others, such as India and Pakistan who engaged in a border conflict in 1999, in greater depth. Thus, the addition of the speech layer helps to capture greater heterogeneity in state positions relative to previous community detection work that focuses on votes alone.

We first embed the speeches into vector space using the Global Vectors for Word Representation (GloVe) algorithm. Word embeddings encode more semantically interesting speech patterns compared to the typical bag-of-words representation of text data [54]. For each year, we utilize the Word Mover’s Distance (WMD) in order to locate distances between states’ speeches [55]. WMD conceptualizes the state-state speech distance problem as one of minimizing the required effort to move one state’s speech embeddings to the vector space location of another state, which we in turn convert to similarity scores [55]. This yields a *V* × *V* speech similarity matrix for each year. Because the resultant vote and speech matrices are densely populated, with each state seemingly connected to every other state, we follow previous work that employs mutual *k*-nearest neighbor graph clustering to yield candidates for multilayer community detection [30, 56]. The notation for the sequence of multilayer weak signal graphs is identical to the bilateral agreements outlined above.

**Fig 1:**
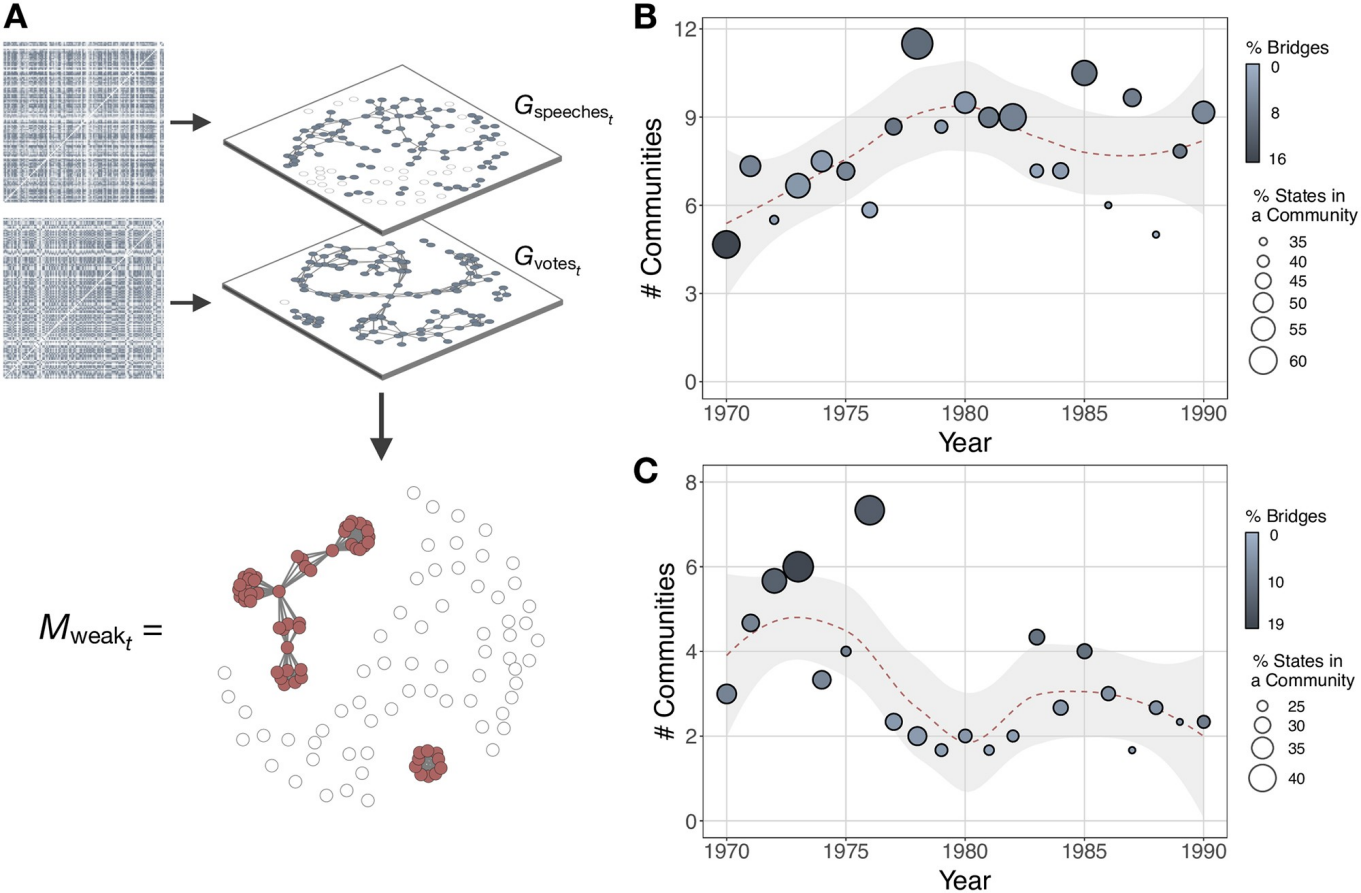
Multilayer community detection procedure. A: mutual 5-nearest neighbor graph clustering on yearly speech (top) and ideal point (bottom) similarity matrices yields candidate adjacency layers for multilayer community detection. Then, we project the edge list recovered from the multilayer extraction algorithm into a single mode network of detected communities. Here, the year 1973 serves as an illustration. The procedure is identical for communities based on cooperation agreements, less the nearest neighbor clustering, since the data are already in adjacency matrix form. B and C: the number of detected communities over time for weak and strong signal communities, respectively. Point weights represent the percentage of states that belong to at least one community. Point shading represents the percentage of states that serve as bridges across at least two communities. Note that these results represent the average of the different preprocessing and parameter settings examined. For ease of trend visualization, the plots include a local weighted regression curve.

With these strong and weak signal candidate layers in hand, we set about detecting multiplex communities. In international politics, different layers might exhibit heterogenous structure. As mentioned, states might initiate bilateral agreements for topic-dependent reasons, and the vote and speech matrices in Fig 1(A) exhibit heterogenous similarity structures. Most community detection methods, however, posit the same community structure across network layers. Therefore, we employ a newly developed method that can accommodate heterogenous structure, namely the Multilayer Extraction procedure [17]. The algorithm identifies densely connected vertex-layers in multilayer networks through a significance-based score that compares the connectivity of an observed vertex-layer set to a fixed degree random graph model. The introductory paper provides technical details [17].

Community detection on yearly instances of strong and weak multilayer networks yields separate sequences of detected community memberships. Single-mode projections of these memberships produce strong and weak multiplex communities for each year, formally $M_{strong}=\left\{M_{strong_{1970}},\ldots ,M_{strong_{t}}\right\}$ and $M_{weak}=\left\{M_{weak_{1970}},\ldots ,M_{weak_{t}}\right\}$, *t* ∈ {1970, …, 1990}, with ties weighted by the number of common communities between two states. The year 1970 represents the beginning of the sequence, because this is the first available year in the corpus of speeches. The year 1990 serves as the final year in the sequence, because previous international conflict research finds evidence that the structural changes associated with the end of the Cold War led to changes in the causal processes that underlie conflict [57]. Thus, we avoid imposing a model that bridges into the post-Cold War era to avoid the conflation of data generating processes. Furthermore, World Treaty Index data availability declines from the 1990s onwards (see [40] page 774).

Fig 1(A) presents the pipeline for the Multilayer Extraction procedure. Fig 1(B) and 1(C) display the number of detected weak and strong signal communities over time, respectively. Point weights indicate the percentage of states that belong to at least one community. Point shading indicates the percentage of nodes that bridge at least two communities. These plots provide a novel glimpse into international polarity with respect to the number of clusters in the system and the ties within and across clusters [58]. Larger points and larger numbers of communities suggest a system in which states are more exhaustively divided into groups (i.e. poles). Lighter points indicate a more modular system with fewer bridging ties (i.e. a system that is more polarized given the constellation of poles). The communities detected from cooperation agreements suggest that states are less exhaustively divided into clusters towards the end of the Cold War, evidenced by a decline in the number of communities and a smaller percentage of states assigned to a community. The communities detected from signals of diplomatic affinity suggest a mean increase in the number of communities over time, with a relatively steady and large percentage of states assigned to a community. Further, greater heterogeneity exists in the weak signal graphs, evidenced by a consistently higher number of communities relative to cooperation agreements over time.

### The emergence of interstate conflict

The detected multiplex communities represent the following. The communities based upon stronger signals represent tightly-knit groups of cooperators, taking into account the relational structure at each layer of the multilayer cooperation network. The communities based upon weaker signals represent clusters of states that exhibit similar expressed preferences in the UN, taking into account the similarity structure in the speech and voting layers. Thus, these multiplex communities provide a useful description of the cooperative mesostructure of the international system.

**Fig 2:**
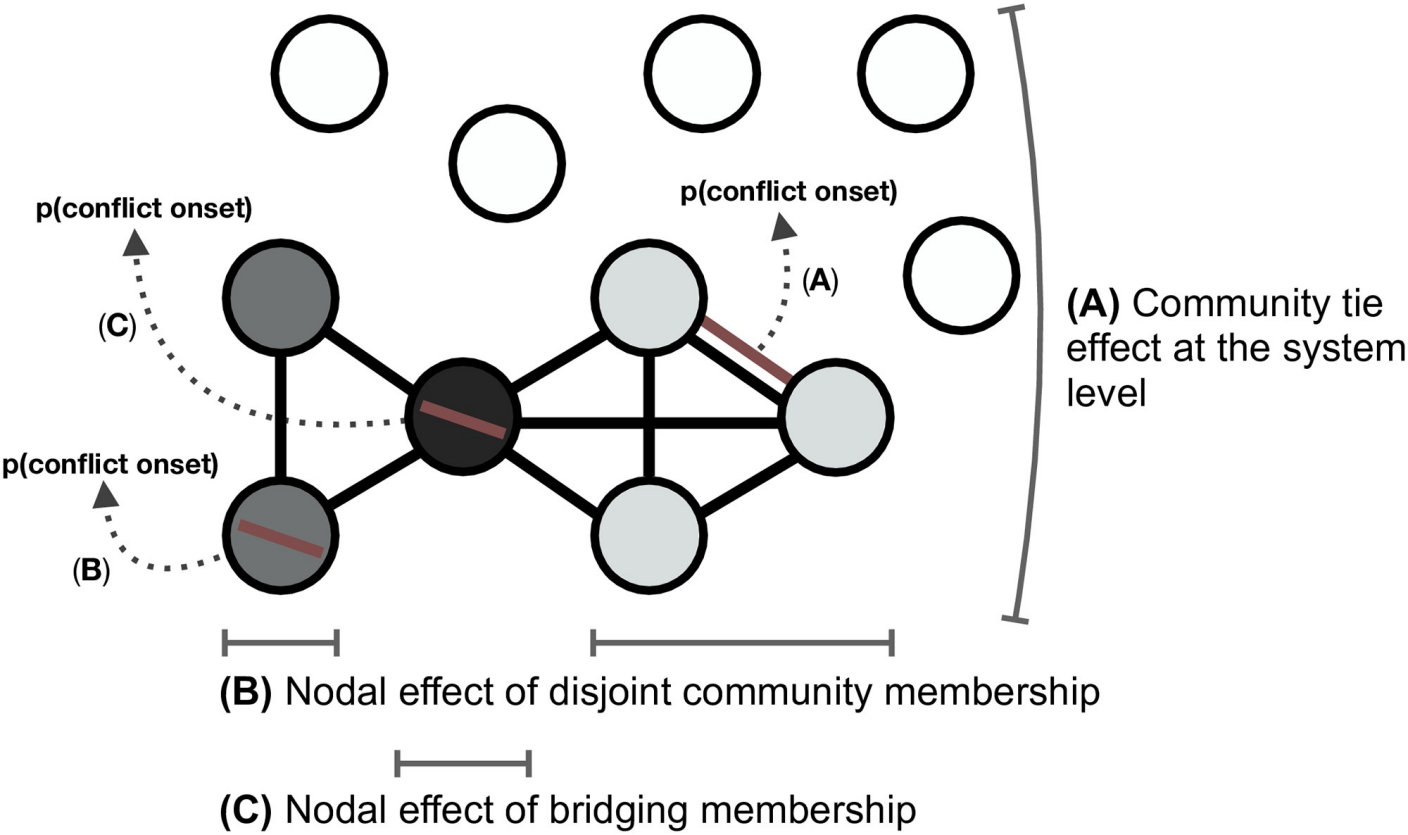
Conflict effects. A: the relationship between a community tie and conflict onset at the system level. B: the effect associated with disjoint community membership and conflict onset, i.e. nodes within the same community that lack membership in other communities. C: the relationship between bridging nodes and conflict onset, i.e. nodes with membership in more than one community.

We now investigate the relationship between these communities and the onset of violent conflict in IR. We first consider the effect of community ties at the system level. Then, we restrict the node set to only the most active states in the system to investigate the ways in which different structural roles within these communities correlate with conflict onset. Fig 2 provides a stylized representation of the tie- and node-level effects under consideration.

As noted in the Introduction, the networked nature of IR often implies a nonindependence of observations that renders logistic regression unsuitable [59]. To circumvent these inferential challenges, we employ a temporal extension to the exponential random graph model [(T)ERGM] [60, 61]. ERGMs are generative models for network data [62], and their results can be interpreted similarly to coefficients from logistic regression: the coefficients provide an estimate for the change in the log-odds likelihood of observing a tie given a one unit change in the independent variable. The outcome network of interest is a yearly snapshot of the conflict onset network. An undirected tie between two states *v*_*i*_ and *v*_*j*_ exists if conflict was initiated in a given year. Model 1 follows a specification by Pauls & Cranmer [30] that contains a battery of covariates traditionally associated with conflict onset. This provides a baseline specification and brings our results into proximity with extant findings. The weak and strong multiplex communities then enter the model as an edge-level covariate in Models 2 and 3, respectively. Table 1 presents these system-level results.

**Table 1:** TERGMs: Analysis of international conflict onset, 1970-1990.

	Model 1 Baseline	Model 2 Weak	Model 3 Strong	Model 4 Strong (No Contig.)
Edges	−**7.76** [−8.03; −7.49]	−**7.72** [−8.09; −7.42]	−**7.69** [−7.98; −7.44]	−**7.38** [−7.68; −7.14]
*Multiplex Comms*.				
Tie Structure		−**0.60** [−1.36; −0.08]	0.01 [−0.26; 0.26]	**0.41** [0.13; 0.73]
*Network Effects*				
Alternating 2-Stars	**1.00** [0.85; 1.13]	**1.04** [0.83; 1.19]	**1.02** [0.86; 1.15]	**0.96** [0.81; 1.09]
4-Cycles	**0.55** [0.46; 0.99]	**0.56** [0.45; 1.13]	**0.54** [0.44; 0.83]	**0.48** [0.39; 0.77]
GWESP (0)	−**0.44** [−1.07; −0.18]	−**0.47** [−5.25; −0.14]	−**0.42** [−1.05; −0.14]	−0.26 [−0.84; 0.05]
*Traditional Covariates*				
Joint Democracy	−0.15 [−0.57; 0.24]	−0.16 [−0.58; 0.22]	−0.13 [−0.56; 0.27]	−**0.77** [−1.30; −0.32]
Direct Contiguity	**3.78** [3.47; 4.15]	**3.65** [3.30; 4.07]	**3.73** [3.43; 4.10]	
Capabilities Ratio	−**0.12** [−0.20; −0.07]	−**0.10** [−0.19; −0.03]	−**0.11** [−0.19; −0.03]	−**0.12** [−0.20; −0.05]
Trade Dependence	−**0.37** [−1.17; −0.06]	−0.26 [−1.10; 0.04]	−**0.39** [−1.25; −0.06]	0.25 [−0.03; 0.40]
Security IGO Dependence	−**0.26** [−0.43; −0.14]	−**0.22** [−0.38; −0.10]	−**0.24** [−0.40; −0.12]	**0.18** [0.07; 0.27]
Economic IGO Dependence	0.00 [−0.02; 0.02]	0.00 [−0.02; 0.03]	−0.01 [−0.03; 0.01]	**0.05** [0.03; 0.08]
Memory (AR, lag = 1)	**2.97** [2.62; 3.31]	**2.97** [2.58; 3.36]	**3.01** [2.64; 3.36]	**4.39** [4.16; 4.66]

Coefficients in bold are significant at or below the *p* = 0.05 level. Confidence intervals in brackets are obtained from 2,000 bootstrapped pseudolikelihood replications. Results represent the average of multiple models fitted using a range of robustness checks.

The coefficient sizes and directions are substantively reasonable. The edges term can be interpreted akin to the intercept term in a logit model. For example, the probability of observing conflict within a given dyad is approximately 0.0004 in Model 1. The significance and coefficient directions of the endogenous network statistics of alternating 2-stars and geometrically weighted edgewise shared partners (GWESP) indicate that conflict tends to cluster within the network. Further, traditional IR covariates display expected signs and effect sizes. For example, two contiguous states display a ceteris paribus 3.78 times higher log odds of conflict onset relative to two non-contiguous states, i.e. an odds increase of 43.82.

In Model 2, the coefficient on ties in weak signal communities is significant and negative. This indicates that conflict is less likely between countries that display strong cohesion in their votes and speeches. Specifically, a given dyad’s log-odds of experiencing conflict decreases by -0.60 for each additional weak signal community tie within that dyad, all else equal. In Model 3, the coefficient on ties in strong signal communities fails to reach significance. This implies that states with ties in the multiplex cooperation network are no more or less likely to engage in conflict than states without cooperative ties. Model 4 presents a more parsimonious specification (i.e. the omission of direct contiguity) in order to examine the effect of these strong community ties if one were to be observed. The omission of direct contiguity is also intuitive to the extent that cooperation agreements encode regional dynamics (e.g. telecommunication agreements often include neighboring countries), and thus the two variables might compete to explain variance. Under this specification, the cooperation community ties become significant and positive. This finding would indicate that a given dyad experiences an increase in the likelihood of conflict given the presence of a cooperation community tie (or ties) within the dyad. Although the absence of contiguity in this model leads us to caution against over-interpretation of this result, the finding is consistent with the absence of discernible conflict suppression effects given the presence of cooperation agreements.

With these system-level results in hand, we next investigate the different structural roles that members serve in these communities. This provides a more granular understanding of the mechanisms through which conflict might emerge and diffuse given the structure of the community. For this analysis, we use the UN as a pivot point and restrict the node set to only those states who voted and delivered a General Assembly speech in a given year. This criteria helps to identify relatively active states in international politics. We note that the results in Table 1 are substantively unchanged by this difference in node set.

Two potential mechanisms are of interest. First, the joint community member effect captures states that are in the same community and no other community. For strong signal communities, these states display the highest levels of cooperative dependency, because they lack ties to states in other communities. For weak signal communities, these states display high levels of intragroup diplomatic affinity and lack appreciable connections to other groups in the UN. Second, the community bridge effect captures states who bridge across more than one community. For strong signal communities, these states are less dependent on any single community but are potentially more vulnerable to conflict due to their exposure to multiple communities. For weak signal communities, these states exhibit relatively pragmatic positions that bridge multiple groups in the UN. Table 2 presents these results.

**Table 2:** TERGMs: Analysis of node effects, 1970-1990.

	Model 5 Weak	Model 6 Strong
Edges	−**7.71** [−8.08; −7.42]	−**7.59** [−8.02; −7.23]
*Node Effects*		
Joint Comm. Member	0.23 [−0.01; 0.46]	−0.04 [−0.26; 0.17]
Comm. Bridge	−0.20 [−0.70; 0.21]	**0.60** [0.26; 1.09]
*Network Effects*		
Alternating 2-Stars	**1.06** [0.81; 1.23]	**1.08** [0.87; 1.23]
4-Cycles	**0.54** [0.44; 1.24]	**0.53** [0.43; 1.05]
GWESP (0)	−**0.46** [−5.24; −0.12]	−**0.45** [−5.20; −0.12]
*Traditional Covariates*		
Joint Democracy	−0.18 [−0.59; 0.17]	−0.24 [−0.70; 0.15]
Direct Contiguity	**3.67** [3.33; 4.12]	**3.72** [3.39; 4.14]
Capabilities Ratio	−**0.11** [−0.20; −0.03]	−**0.12** [−0.21; −0.05]
Trade Dependence	−0.29 [−1.25; 0.03]	−**0.41** [−1.50; −0.02]
Security IGO Dependence	−**0.23** [−0.40; −0.10]	−**0.15** [−0.29; −0.04]
Economic IGO Dependence	−0.00 [−0.03; 0.02]	−0.02 [−0.04; 0.01]
Memory (AR, lag = 1)	**2.78** [2.43; 3.14]	**2.84** [2.32; 3.29]

Coefficients in bold are significant at or below the *p* = 0.05 level. Confidence intervals in brackets are obtained from 2,000 bootstrapped pseudolikelihood replications. Results represent the average of multiple models fitted using a range of robustness checks.

For weak signal communities, the results presented in Model 5 indicate a lack of effect for both joint community members and community bridges. This implies that weak community members are no more or less likely to engage in conflict with each other and that bridges are no more or less likely to experience conflict. For strong signal communities, the results of Model 6 suggest a lack of joint community member effect but a significant and positive relationship between conflict and strong community bridges. This implies that states who bridge multiple communities are more likely to experience conflict and perhaps provide a pathway through which conflict might diffuse across communities.

## Discussion

The above results represent the first evidence on the relationship between multiplex communities and the onset of international conflict beyond previous attention to the Kantian triad (see [29]). For communities detected across layers of UN votes and speeches, the results confirm and extend the finding of a previous study based upon voting behavior alone: diplomatic cohesion appears to negatively associate with conflict in the international system [30]. Although the result is substantively similar, the addition of the speech layer provides useful information on the expressed preferences of states that is otherwise absent in roll call data alone.

The communities detected across layers of cooperation agreements present a more challenging picture. The most optimistic model specification yields a lack of association between community ties and conflict onset. A more pessimistic specification yields a positive association between cooperative ties and conflict onset. This result is surprising, because cooperation and conflict are often thought to display an inverse relationship, see e.g. [22, 63]. At least two mechanisms might explain this result. First, states at times employ bilateral agreements to manage contentious issues [64]. When agreements fail, this tie could provide an indicator for potential conflict onset. Second, those states who interact more often or are most active in agreement formation might face greater opportunities for disputes to arise. Similar arguments have been made in the case of alliance formation and geographically contiguous dyads [65–67]. For example, Traag & Bruggeman [13] uncover a similar result in the assessment of their detection algorithm on alliance data, namely that conflict tends to emerge within detected communities. As Waltz ([20] page 138) pointed out, “[i]t is impossible to get a war going unless the potential participants are somehow linked.” Either way, this finding calls into question the extent to which cooperators enjoy more peaceful outcomes than non-cooperators.

This communities and conflict puzzle is in part empirically explained by attention to structural roles within communities. Conflict diffusion via network ties is a well-established pattern in IR [68]. This study augments this finding: states that bridge cooperative communities are especially conflict prone, and this bridge points to a path through which conflict might diffuse to clusters of states. Those states with exclusive membership in a single community, however, are no more or less likely to engage in conflict with community members. This finding reiterates the open question surrounding interdependence and conflict. Further, this study finds scant evidence that community roles in the UN explain meaningful variance in conflict outcomes: states exclusively aligned with a single bloc and states who pragmatically bridge multiple communities enjoy no detectable change in conflict likelihood. This finding suggests that community membership is more important for conflict outcomes than the specific role that countries serve within communities in the UN.

Taken together, these results suggest at least two implications. First, for IR cooperation research, increases in tie density do not necessarily lead to decreased levels of conflict. Indeed, previous network science findings indicate that cooperative stability requires enough structure to support cooperation but not so much as to stifle it [32]. Second, for network cooperation research, future work could more rigorously explicate the theoretical mechanisms through which cooperation might suppress conflict. Cooperators tend to cluster on graphs [69]. The above analysis suggests that conflict might diffuse via nodes that bridge these clusters, which could paradoxically increase the likelihood that community members face conflict. Nonetheless, this study’s results reiterate the present paucity of observational findings on the relationship between communities and outcomes in social systems. Domain-specific empirical applications will help to narrow the scope of this problem whilst shedding light on the utility of new detection algorithms for questions of computational social science interest.

## Materials and methods

### Data

As described above, we utilize bilateral cooperation agreements and United Nations (UN) votes and speeches in order to construct the strong and weak signal multilayer graphs, respectively. We obtain the former from the World Treaty Index [38, 39], which provides the most complete record of bilateral agreements in international relations (IR). These data represent a rich source of information about international cooperation (see e.g. Kinne [40]) and have previously been used to operationalize peaceful relations between countries (see e.g. Kasten [70]). We specifically include the treaties under the categories of “Science and Technology” (7SCIEN), “Military Procedures” (9MILIT), “Raw Materials Trade” (3COMMO), “Fisheries” (8FISH), and “Telecommunications” (6TELCO). The dataset contains an edge list of dyads that are party to the treaty, as well as the year that the treaty was signed and a qualitative description of the treaty’s purpose.

For the weak signal data, we employ UN votes and UN General Debate speeches. For roll call data, we utilize yearly country ideal points estimated on a single dimension via a dynamic ordinal spatial model [48]. This model provides a unidimensional reduction of countries’ yea, nay, or abstain decisions on a variety of UN agenda voting items, often interpreted in political science to be a useful indication of a country’s expressed preferences or positions with respect to a given topic. The employment of these ideal points helps to avoid the issues posed by the high levels of voting similarity in the UN when attempting to detect communities, as identified in Macon et al [49]. Furthermore, in contrast to more common unipartite projections of bipartite graphs based on similarity measures (see e.g. Yildim & Coscia [71]), the ideal points are based on a more explicit theoretical model of vote choice given a state’s preferences (see Bailey et al [48]). These data are available online at Harvard Dataverse: hdl:1902.1/12379. In addition, we utilize the record of annual speeches delivered by country representatives—predominantly heads of state or government—during the annual UN General Debate [44]. These speeches are stored as plain text files with associated metadata and are available online at Harvard Dataverse: doi.org/10.7910/DVN/0TJX8Y.

The paper’s main text describes the vote and speech similarity measures that we employ. In order to move from similarity matrices to candidate adjacency matrix layers for multilayer community detection, we utilize a mutual *k*-nearest neighbor graph approach (see e.g. Ozaki et al [56]). We employ the mutual *k*-nearest neighbor graph approach in order to ensure that our replication procedure follows closely the original clustering procedure of Pauls & Cranmer [30], such that any differences in results can be attributed to the addition of the speeches layer in the multilayer setting. For useful discussions about backboning methods and graph sparsification, see e.g. Serrano et al [72], Slater [73], and Zhang et al [74].

After the performance of community detection on the strong and weak signal graphs, we model the detected communities against the onset of violent conflict in IR. We utilize data from a previous study by Pauls & Cranmer [30] that looked at a similar question as the current study, and we thank the authors for sharing these materials. The outcome network of interest is constructed from conflict onset data from the Correlates of War (COW) project’s Militarized Interstate Dispute (MID) dataset (v4.1) [75]. An undirected tie is considered to be present if a MID of level 4 or 5 was initiated between a dyad during the year of interest. These are the two levels of greatest hostility covered in the dataset, with the former corresponding to such actions as occupation of territory or declaration of war, and the latter corresponding to the initiation of war. More details on the conflict data are available online at the Inter-University Consortium for Political and Social Research: doi.org/10.3886/ICPSR24386.v1.

The inferential model also includes the following covariates. Democracy is a node attribute equal to 1 if the country’s Polity IV score is greater than or equal to 7. Direct contiguity enters the model as an indicator variable equal to 1 if two countries share a geographic border or share a sea border within 400 miles of each other. Capabilities ratios capture the ratio of two countries’ Composite Index of National Capabilities scores, which utilizes various measures of state capabilities, including population, military expenditures, and iron and steel production. Trade dependence is operationalized as the total yearly trade flow from *v*_*i*_ to *v*_*j*_, divided by the GDP of *v*_*i*_. Finally, security and economic IGO dependence are operationalized as the total number of third-party states to which *v*_*i*_ and *v*_*j*_ are jointly connected through security and economic-oriented intergovernmental organizations, respectively. Pauls & Cranmer [30] provide more details on these variables.

### Models

To locate vector space representations of the corpus, we utilize the Stanford NLP group’s Global Vectors for Word Representation (GloVe) unsupervised learning algorithm [54]. GloVe is a popular log bilinear, weighted least squares model that trains on global word-word co-occurence counts to make efficient use of the corpus statistics. Because it factorizes a word-context co-occurrence matrix, it shares affinities with traditional count methods like latent semantic analysis or principle component analysis. First, the raw texts are stemmed and trimmed of any tokens that appear fewer than 5 times or in fewer than 5% of speeches across the corpus. This pre-processing was found to improve the quality of the located embeddings. We use a context window of 5 (i.e. 5 words before and 5 words after the target feature). To tune the model’s parameters, we fit the model to word vectors of size 50, 100, and 200 with maximum term co-occurrences of 15 and 25 for the weighting function. This yields “main” and “context” vectors which are subsequently averaged together per the suggestion of the original GloVe paper [54] to locate the final embedding space.

We then calculate the distances between each pair of states in each year using the relaxed variant of the Word Mover’s Distance (RWMD) [55]. This measure utilizes the embedding space and each country’s term-document matrix to measure the cumulative distance required to transform one state’s speech point cloud into that of another state. This procedure helps to ensure that distances are not simply a function of the use of different words, but rather differences in the semantic structure of two countries’ speeches. The SI presents more details on this procedure. We use the quanteda package [76] for corpus ingestion, and the text2vec package [77] for fitting the GloVe models and calculating the RWMDs. All analysis is conducted in the R statistical programming environment [78].

To model the evolution of the conflict onset network, we employ a temporal extension to the exponential random graph model [(T)ERGM] [60, 61]. Originally proposed by Wasserman & Pattison [62] (and also known as p* models), ERGMs are generative models for the performance of inference on network data that have found widespread employment across the network and social sciences [79–81]. The model used here assesses uncertainty using a bootstrap approach proposed by Desmarais & Cranmer [82, 83], and the models were fitted using the btergm package [84] in the R statistical programming environment [78]. Regarding interpretation, our results speak to the likelihood of conflict between two states *v*_*i*_ and *v*_*j*_ given the intensity of cooperation between *v*_*i*_ and *v*_*j*_. We do not extrapolate these results further, such as the likelihood of conflict between *v*_*i*_ and some third party state *v*_*k*_ given the cooperative activity of *v*_*i*_ and *v*_*j*_. At the same time, the results do permit the conclusion that highly active states—i.e. states with several community ties—would experience changes in the likelihood of conflict onset commensurate with the number of community ties. See Desmarais & Cranmer [85] for more information about the interpretation of ERGMs with respect to various levels of the network.

In addition to the variables outlined above, we specify the following variables in the model. The edges term represents the total number of ties in the graph, akin to the intercept term in regression models. Alternating 2-stars adds alternating sequences of two-paths (i.e. unclosed triangles) to the model, and 4-cycles captures the existence of four nodes connected in a box-like structure, namely *e*_*iv*_ = *e*_*iu*_ = *e*_*jv*_ = *e*_*uj*_ = 1 [86]. Finally, geometrically weighted edgewise shared partners (GWESP) adds a statistic equal to the geometrically down-weighted shared partner distribution, here with a fixed decay parameter of 0. The latter three of these statistics capture potential clustering in the conflict onset network. The community detection results depend on a number of choices surrounding data representation and parameter selection, such as the hyperparameters for the embedding model and the proportion of vertices used to initialize the Multilayer Extraction algorithm. To enhance robustness, we conduct the analysis using the different GloVe hyperparameters described above, as well as vertex initialization proportions of .20, .25, and .30 during the Multilayer Extraction procedure for the strong signal graphs. The results presented in the paper’s Emergence of Interstate Conflict section represent the mean results of these analyses.

## Supporting information

S1 Text (PDF)

S1 Table Nearest features based on cosine similarity. (PDF)

S1 Fig t-SNE projection. (PDF)

S2 Fig WMD abstract example. (PDF)

S3 Fig Models 1 and 2 in-sample goodness-of-fit. (PDF)

S4 Fig Models 3 and 4 in-sample goodness-of-fit. (PDF)

S5 Fig Models 5 and 6 in-sample goodness-of-fit. (PDF)

S6 Fig Test set predictive accuracy. (PDF)
