# Supplementary material for: Multiplex communities and the emergence of international conflict
Source: PLoS One. 2019 Oct 16;14(10):e0223040. doi: 10.1371/journal.pone.0223040 (PMC6795412; doi:10.1371/journal.pone.0223040)
Supplement: S1 Text — (PDF) [file pone.0223040.s001.pdf]

# Supporting information for: Multiplex communities and the emergence of international conflict

Caleb Pomeroy[1,*], Niheer Dasandi[2], and Slava Jankin Mikhaylov[3]

[1]Department of Political Science, The Ohio State University, Columbus, Ohio, United States

[2]School of Government, University of Birmingham, Birmingham, United Kingdom

[3]Data Science Lab, Hertie School, Berlin, Germany.

[*]pomeroy.38@osu.edu

## The UN General Debate Corpus

We draw on the newly released *UN General Debate Corpus* [1] which contains every country statement in the UN General Debate between 1970 and 2017. The General Debate (GD) takes place every September at the start of each new session of the UN General Assembly (UNGA). It provides all member states with the opportunity to address the UNGA and to present their perspective on key issues in world politics. Governments use their GD statements to put on the record their position on events that have occurred during the past year and on longer-term underlying issues in world politics related to issues such as conflict, terrorism, development, human rights, and climate change.

A principal difference between GD statements and UNGA voting is that the GD statements are not institutionally connected to decision-making in the UN. As a result, governments are free to discuss the issues they consider to be of greatest importance in world politics, regardless of whether an issue is on the formal agenda of the UNGA. Therefore, as Smith [2] notes, the General Debate acts "as a barometer of international opinion on important issues, even those not on the agenda for that particular session." In providing information about states' preferences on world politics, the GD provides a valuable data source for measuring polarization in International Relations. In addition to being the one major forum where states present their views on international politics free from external constraints, the fact that it takes place annually and includes all UN member states enables comparison over time and across countries. Readers are encouraged to consult Baturo et al [1] for a comprehensive introduction to the corpus.

As stated in the main text, we discuss an example where disagreement is obvious in states' GD speeches but less obvious in their voting behavior. Consider the following brief excerpts from the GD speeches of Greece and Turkey in 1974.

> **Greece:** On 15 July a coup, condemned by all of us, was staged to overthrow Archbishop Makarios, the legitimate, elected President of the Republic. This coup was not directed against the Turkish Cypriot community of the island... During the fighting while the coup was in progress, not a single Turkish Cypriot was killed or injured. Yet five days later, large Turkish invasion forces were landing in Cyprus and the Turkish Air Force was launching indiscriminate attacks against unarmed civilians, under the flimsy pretext of protecting the Turkish Cypriot minority on the island, which, I repeat, had not been harmed in any way... Two hours later, the Turkish troops were on the move again, sowing death and destruction, killing United Nations troops, bombing hospitals and schools. Repeated cease-fire calls by the Security Council went unheeded. Turkey

even ignored the ceasefire proclaimed by its own Prime Minister on 16 August 1974.

**Turkey:** Turkey has constantly had to face faits accomplis of increasingly serious scope, particularly since 1963. The most recent and the most serious of these faits accomplis was, as we all know, that of 15 July last: a foreign Power undertook a coup d'etat which it had long been fomenting and the purpose of which was to annex the island... The coup d'etat of 15 July was directed precisely against the Turkish community and was directly aimed at the annexation of the island to Greece... I have not, however, finished correcting all the false allegations and baseless charges made by my colleague. I reserve the right to do so when we speak on this matter before the General Assembly. My Greek colleague's speech, unfortunately, shows the nature of the atmosphere in which the debate will take place on the future of the two communities, Turkish and Greek, in the island.

The two representatives are outlining their positions on the controversy related to the Turkish invasion of Cyprus. Expressed disagreement on this topic is clearly present in the speeches, but as mentioned in the main paper, the two countries' voting ideal points for that year are the most similar amongst all NATO members. A further example is illustrated in the speeches and voting habits of India and Pakistan in 1999, the year the two countries went to war (the Kargil War). Consider the following excerpts from their General Debate statements that year:

**Pakistan:** The Kargil crisis was a manifestation of the deeper malaise spawned by the unresolved Kashmir problem and India's escalating repression of the Kashmiri people. India launched a massive military operation in Kargil and threatened a wider conflict by mobilising its armed forces all along the Pakistan-India international border. Pakistan acted with restraint... India's repression in Jammu and Kashmir has killed thousands of Kashmiris, forced hundreds of thousands into exile, led to three wars between Pakistan and India and consigned the two countries to a relationship of endemic conflict and mistrust.

**India:** Premeditated aggression by regular forces was committed against India. Not simply was the Lahore Declaration violated, but so was the Simla Agreement, which had prevented conflict for more than a quarter of a century. In self-defence, yet with the utmost restraint, India took all necessary and appropriate steps to evict the aggressor forces from its territory.... We have been greatly disappointed by this compulsive hostility of Pakistan, because it is an aberration in our region today, where all the other South Asian Association for Regional Cooperation (SAARC) countries are at peace with each other, and are trying, bilaterally and through the SAARC mechanisms, to tackle together the great challenge of development.

Tensions are clearly present in the textual data of the respective countries. That same year, however, India and Pakistan casted very similar votes in the UN, with ideal points of -0.797 and -0.739, respectively. Therefore, both sources of data appear to provide useful signals of different aspects of underlying state preferences.

## Word embeddings

In order to use texts together with votes to estimate position affinity, we first consider how to better exploit the information contained in textual data, namely unsupervised learned word embeddings. In the broader natural language processing (NLP) literature, there has been a surge of research devoted to the development of distributional representations of speech which retain syntactical language qualities in ways that the bag-of-words (BOW) approach typically used in political text analysis research is not equipped to retain. The hypothesis claims that words that occur in similar contexts tend to have similar meanings [3]. When operationalized, the unique intuition is that similar words and phrases, such as *"atomic, weapons"* and *"nuclear, warheads"* are found in relatively proximate vector space locations. Although the BOW performs surprisingly well, this example has no features in common, and a BOW representation would assign low similarity scores or high distances. Word embeddings help to ensure that communities are detected amongst states that are actually expressing different positions, as opposed to simply using different language to express the same sentiment.

When results are projected onto a two dimensional surface, language relationships surface, such as the clustering of synonyms, antonyms, scales (e.g. *democracy* to *authoritarianism*), hyponym-hypernyms (e.g. *democracy* is a type of *regime*), co-hyponyms (e.g. *atomic bombs* and *ballistic missiles* are types of *weapons*), and groups of words which tend to appear in similar contexts like *diplomat*, *envoy*, and *embassy*. Mikolov and collaborators introduce an evaluation scheme based on word analogies that examines dimensions of difference in vector space [4, 5]. They originally reached the surprising conclusion that simple vector addition and subtraction uncovers interesting linear substructures of human language, famously that $\mathsf{king} - \mathsf{man} + \mathsf{woman} = \mathsf{queen}$.

To locate vector space representations of our corpus, we utilize the Stanford NLP group's Global Vectors for Word Representation (GloVe) unsupervised learning algorithm [6]. GloVe is a popular log bilinear, weighted least squares model that trains on global word-word co-occurence counts to make efficient use of the corpus statistics. Because it factorizes a word-context co-occurrence matrix, it is closer to traditional count methods like latent semantic analysis or principle component analysis.[1] Readers are encouraged to consult the GloVe paper for full technical details, but we describe our approach and resultant vector space here. The model is expressed as:

$$J(\theta) = \frac{1}{2}\sum_{i,j=1}^{W} f(P_{ij})(u_i^T v_j - \log P_{ij})^2 \tag{S1}$$

where $\theta$ represents parameters, $W$ is the vocabulary size, $u \in R^d$ and $v \in R^d$ are column and row word vectors, $P_{ij}$ is the co-occurrence matrix of all pairs of words that ever co-occur, and $f(\cdot)$ is a weighting function which assigns lower weights to words that frequently co-occur. This lattermost term serves as a cap on very frequent words, for example articles like "the" which provide little predictive information. The algorithm seeks to minimize the distance between the inner product of the word vectors and the log count of the co-occurrence of the two words. Compared to skip-gram approaches which update at each context window, it is clear from the utilization of $P_{ij}$ that the model trains relatively quickly since it uses the known corpus statistic of word co-occurrences for the entire corpus at once. To our knowledge, this is one of the first IR applications to use word embeddings (see also [8]).

[1] For recent reviews of the distributional semantics literature, see Turney & Pantel [3] and Lenci [7].

The Models section of the main paper outlines the parameters we chose.[2] We follow the computer science literature suggestion of tuning these parameters until reasonable and reliable linear combinations of language are located. Future work should explore in greater detail how systematic tuning decisions for social science applications can be made. Here, we present various qualitative checks on the located embeddings. First, we consider the analogical performance of various features:

$$\begin{aligned}
\vec{v}(\text{“peac-”}) - \vec{v}(\text{“agreement”}) + \vec{v}(\text{“weapon”}) &= \underset{.64}{\vec{v}(\text{“nuclear”})}\,,\ \underset{.62}{\vec{v}(\text{“destruct”})} \\
\vec{v}(\text{“west”}) - \vec{v}(\text{“nato”}) + \vec{v}(\text{“russia”}) &= \underset{.57}{\vec{v}(\text{“east”})}\,,\ \underset{.50}{\vec{v}(\text{“pakistan”})} \\
\vec{v}(\text{“terrorist”}) + \vec{v}(\text{“bomb”}) &= \underset{.82}{\vec{v}(\text{“attack”})}\,,\ \underset{.63}{\vec{v}(\text{“barbar-”})} \\
\vec{v}(\text{“environment”}) + \vec{v}(\text{“pollut-”}) &= \underset{.74}{\vec{v}(\text{“degrad-”})}\,,\ \underset{.65}{\vec{v}(\text{“ecolog-”})}
\end{aligned} \tag{S2}$$

where each $\vec{v}$ describes a vector space location of the given feature, and the cosine similarity between each vector space location is added or subtracted to find the closest vector offsets (with cosine similarity printed underneath). These analogies are interpreted, for example, as “agreement” is to “peace” as “weapon” is to “destruct”. These examples appear to encode relations of cause-effect and geographic alliance patterns, respectively. The latter two are not analogies, but rather the resultant vector space location when the first two vectors are added together. Although these linear combinations look quite reasonable, it is worth checking the nearest neighbors (as measured by cosine similarity) of various features in the embedding space. These are listed in Table S1.

As with the analogical examples, it appears in Table S1 that reasonable groupings of words are detected. Finally, to get a sense of the semantic structure of the embedding space, we plot the 200 nearest words to the vector space of “weapon” and project this onto two dimensions using the common t-SNE algorithm.

Fig S1 shows quite intuitive clustering of features. For example, in the third quadrant exists a cluster of features that generally relate to the industrial aspects of weaponry, evidenced by terms like “manufactur-,” “produc-,” and “export.” In quadrant two we see a geographic clustering with terms like “asia,” “europ-,” and “america.” Finally, in the bottom right of quadrant four exists a clustering of terms commonly associated with the international regulation and governance of weaponry, with feature like “prohibit,” “chemic-,” and “biolog-.” These qualitative checks increase our confidence that the located embedding space should contain useful information about the speeches delivered by states. As found in the wider NLP literature, the implication is that these vector space models are surprisingly effective at capturing different lexical relations, despite the lack of supervision.

To measure expressed (dis)agreement in these speeches, it is necessary to derive a document-level representation of the learned embeddings. Although well-established measurements based on cosine similarity, Euclidean distance, or Pearson correlations could be applied to the word embeddings, we utilized the relaxed variant of a relatively newly introduced document distance measure that exploits information contained in both the word embeddings and term-document matrices: the (relaxed) Word Mover’s Distance [(R)WMD] [10]. WMD measures the cumulative distance required to transform one state’s speech point cloud into that of another state,

[2] As with similar machine learning tasks, we note that our results are sensitive to choices of hyperparameter settings and random seeds (see e.g. Henderson et al [9]).

ensuring that differences do not simply reflect the use of different words. States employ varied language and lexical patterns to describe similar topics. For example, if state A says “nuclear weapons are bad," and state B says “atom bombs are terrible," the only feature in common is the term “are," which leads to near-orthogonality in their BOW vectors and low similarity scores. If a third state C says “atom bombs are good," then B and C would exhibit the highest cosine similarity of the three, despite having the opposite expressed policy positions. Fig S2 shows an illustration of the motivation behind this distance metric, which has been shown to yield state-of-the-art classification accuracy [11].

Although WMD is relatively fast to compute, we use the relaxed variant (RWMD), which results in tighter bounds and is shown to yield lower test error rates. In short, this relaxes the optimisation problem through the removal of one of the two constraints. If we let $\mathbf{d}$ and $\mathbf{d}'$ be the BOW representations of two documents in the $n-1$ dimensional simplex of word distributions which we obtained above, we can express RWMD as:

$$\min_{\mathbf{T}\geq 0} \sum_{\mathrm{i,j}=1}^{\mathrm{n}} \mathbf{T}_{\mathrm{ij}}\mathrm{c(i,j)} \qquad \text{s.t.} \quad \sum_{j=1}^{n} \mathbf{T}_{ij} = d_i \quad \forall i \in \{1,...,n\}. \tag{S3}$$

where $\mathbf{T} \in R^{n\times n}$ is a sparse flow matrix where $\mathbf{T} \geq 0$ denotes how much of word $i$ in $\mathbf{d}$ travels to word $j$ in $\mathbf{d}'$ and $\sum_{\mathrm{i,j}=1}^{\mathrm{n}} \mathbf{T}_{\mathrm{ij}}\mathrm{c(i,j)}$ represents the distance between the two documents, i.e. the cost of moving all words from $\mathbf{d}$ to $\mathbf{d}'$. Then, the optimal solution is found when each word in $\mathbf{d}$ moves all of its probability mass to the most similar word in $\mathbf{d}'$. This optimal matrix $\mathbf{T}^*_{ij}$ is decided by:

$$\mathbf{T}^*_{ij} = \begin{cases} d_i & \text{if } j = \mathrm{argmin_j} c(i,j) \\ 0 & \text{otherwise.} \end{cases} \tag{S4}$$

where $d_i$ is the distance of interest which we normalize and convert to a similarity score. The result is a list of $V \times V$ matrices $\mathbf{A}$ with one matrix $A_t$ for each year in the corpus and where $A_{ij}$ is the speech similarity score between states $i$ and $j$ with the diagonals of the matrices set to 0. The original paper provides further details [10].

## Model evaluations

In order to compare the fits of the models reported in the main body of the paper, we also present the in-sample fit and out-of-sample performance of each model. For ERGMs, in-sample goodness-of-fit is assessed through the simulation of several networks using the fitted model. Then, the analyst measures how well the simulations capture network statistics that were not originally specified in the model. For each time step, 50 networks were simulated from the fitted model and statistics for degree, edge-wise shared partners, and modularity are plotted below. If the model has sufficiently captured the data generating process (i.e. the onset of violent conflict), then the statistics from the simulated networks (represented as box plots) should be near to the distributions of those statistics in the observed network (represented as black lines). Ideally, the black lines would cross the medians of the simulated box plots. These results are presented in Fig S3 through S5, with values for degree and edge-wise shared partners offset by 1 and logged in order to aid in visualization. The simulations from each of the models display quite strong goodness-of-fits. Although neither model drastically outperforms the other, this increases our

confidence that the data generating process has been adequately captured.

Second, although the prediction of conflict out-of-sample is not an objective of this paper, it is worthwhile to compare the models' relative test set accuracies. This more challenging task helps to assess the extent to which one model over another more adequately captures the data generating process of conflict onset, and addresses the issue of overfitting on the training set. For ERGMs, an especially useful metric is the area under the precision recall curve (AUCPR). For this task, a fitted model is trained on five year windows and the sixth year is taken as a test set for out-of-sample conflict onset prediction (i.e. the formation of a tie in the outcome network of interest). The box plots in Fig S6 display the AUCPR for each of the reported models in the main paper, as well as the performance of a random graph for comparison.

These results suggest that the six models display quite similar out-of-sample predictive performance. Therefore, we find scant evidence for an argument to prefer one model over the other. Instead, the specification likely comes down to theoretical motivations and the research question at hand. Although these model are built for inference, and not prediction, this is further confirmation that conflict onset prediction indeed remains a challenging task for statisticians and political scientists. The in-sample goodness-of-fits, however, indicate that the model is adequately specified to the data at hand.

## References

[1] A. Baturo, N. Dasandi, and S. Jankin Mikhaylov, “Understanding state preferences with text as data: Introducing the UN general debate corpus,” *Research and Politics*, vol. 4, no. 2, pp. 1–9, 2017.

[2] C. Smith, *Politics and Process at the United Nations: The Global Dance*. Boulder, CO: Lynne Rienner, 2006.

[3] P. D. Turney and P. Pantel, “From frequency to meaning: Vector space models of semantics,” *Journal of Artificial Intelligence Research*, vol. 37, pp. 141–188, 2010.

[4] T. Mikolov *et al.*, “Distributed representations of words and phrases and their compositionality,” in *Advances in Neural Information Processing Systems*, pp. 3111–3119, 2013.

[5] T. Mikolov *et al.*, “Efficient estimation of word representations in vector space,” *arXiv preprint arXiv:1301.3781*, 2013.

[6] J. Pennington, R. Socher, and C. D. Manning, “GloVe: Global vectors for word representation.,” in *Empirical Methods in Natural Language Processing (EMNLP)*, vol. 14, pp. 1532–1543, 2014.

[7] A. Lenci, “Distributional models of word meaning,” *Annual Review of Linguistics*, no. 0, 2017.

[8] A. M. Lauretig, “Identification, interpretability, and bayesian word embeddings,” *arXiv preprint arXiv:1904.01628*, 2019.

[9] P. Henderson, R. Islam, P. Bachman, J. Pineau, D. Precup, and D. Meger, “Deep reinforcement learning that matters,” in *Thirty-Second AAAI Conference on Artificial Intelligence*, 2018.

[10] M. Kusner, Y. Sun, N. Kolkin, and K. Weinberger, “From word embeddings to document distances,” in *International Conference on Machine Learning*, pp. 957–966, 2015.

[11] G. Huang, C. Guo, M. J. Kusner, Y. Sun, F. Sha, and K. Q. Weinberger, “Supervised word mover’s distance,” in *Advances in Neural Information Processing Systems (NIPS)*, pp. 4862–4870, 2016.
