# Supplementary material for: Multiplex communities and the emergence of international conflict
Source: PLoS One. 2019 Oct 16;14(10):e0223040. doi: 10.1371/journal.pone.0223040 (PMC6795412; doi:10.1371/journal.pone.0223040)
Supplement: S1 Table — (PDF) [file pone.0223040.s002.pdf]

	**war**	**law**	**trade**	**west**	**human**	**nuclear**
1	cold	rule	market	east	right	weapon
2	end	norm	commod	north	life	prolifer
3	conflict	principl	product	south	protect	test
4	confront	legal	export	asia	digniti	chemic
5	violenc	intern	industri	bank	valu	destruct
6	fratricid	right	monetari	asian	fundament	arsenal
7	horror	respect	restrict	gaza	justic	arm
8	devast	justic	develop	africa	law	treati
9	destruct	regul	econom	europ	and	disarma
10	after	fundament	system	southern	individu	armament

Table S1: *Nearest features based on cosine similarity.* Top 10 nearest features in vector space to target feature in column header based on cosine similarity.
