# Supplementary material for: Multiplex communities and the emergence of international conflict
Source: PLoS One. 2019 Oct 16;14(10):e0223040. doi: 10.1371/journal.pone.0223040 (PMC6795412; doi:10.1371/journal.pone.0223040)
Supplement: S1 Fig — (PDF) [file pone.0223040.s003.pdf]

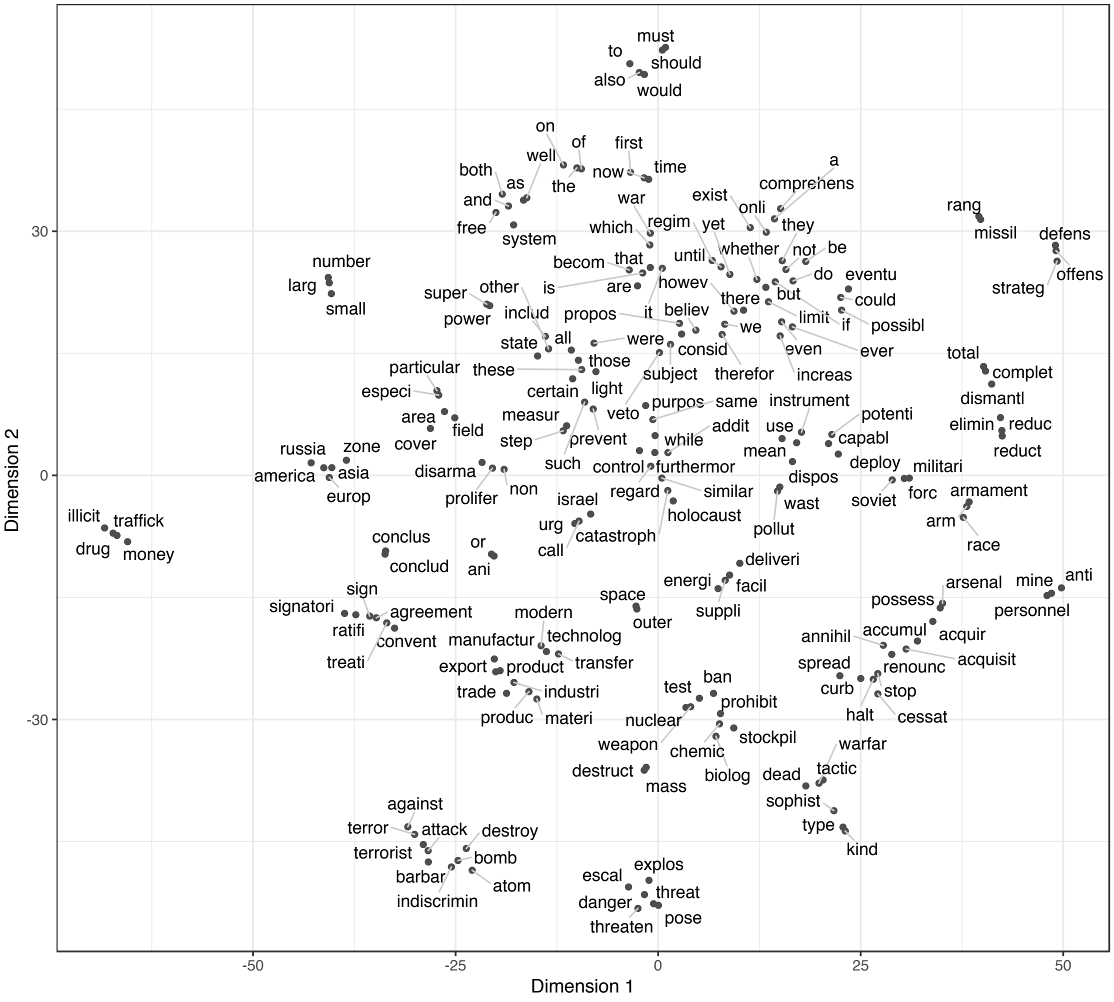

Figure S1: *t-SNE Projection.* 200 nearest words to the vector space of “weapon”, projected onto two dimensions using the t-SNE algorithm.
