# Supplementary material for: Multiplex communities and the emergence of international conflict
Source: PLoS One. 2019 Oct 16;14(10):e0223040. doi: 10.1371/journal.pone.0223040 (PMC6795412; doi:10.1371/journal.pone.0223040)
Supplement: S2 Fig — (PDF) [file pone.0223040.s004.pdf]

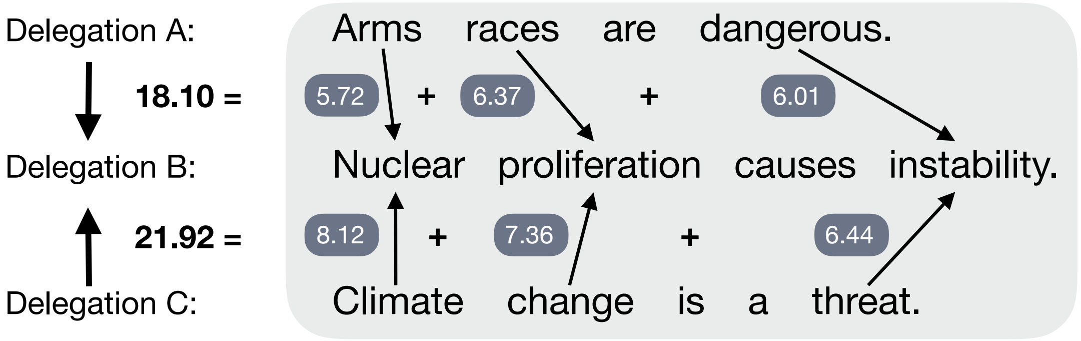

Figure S2: *WMD Abstract Example.* Three strings of text serve as an example of the motivation behind the employment of WMD. Delegations A and B both discuss issues surrounding international armament but utilize different language. This leads to very low similarity scores under a bag-of-words framework. WMD innovates by capturing the distance required to move one document to the vector space location of another document. In this example, although all three delegations discuss issues relevant to security, the speeches of delegations A and B are nearer in vector space than the speeches of delegations B and C. Actual Euclidean distances from our estimated embeddings are used for illustration. Adapted from Fig 2 in Kusner et al [10].
