# Supplementary material for: Multiplex communities and the emergence of international conflict
Source: PLoS One. 2019 Oct 16;14(10):e0223040. doi: 10.1371/journal.pone.0223040 (PMC6795412; doi:10.1371/journal.pone.0223040)
Supplement: S4 Fig — (PDF) [file pone.0223040.s006.pdf]

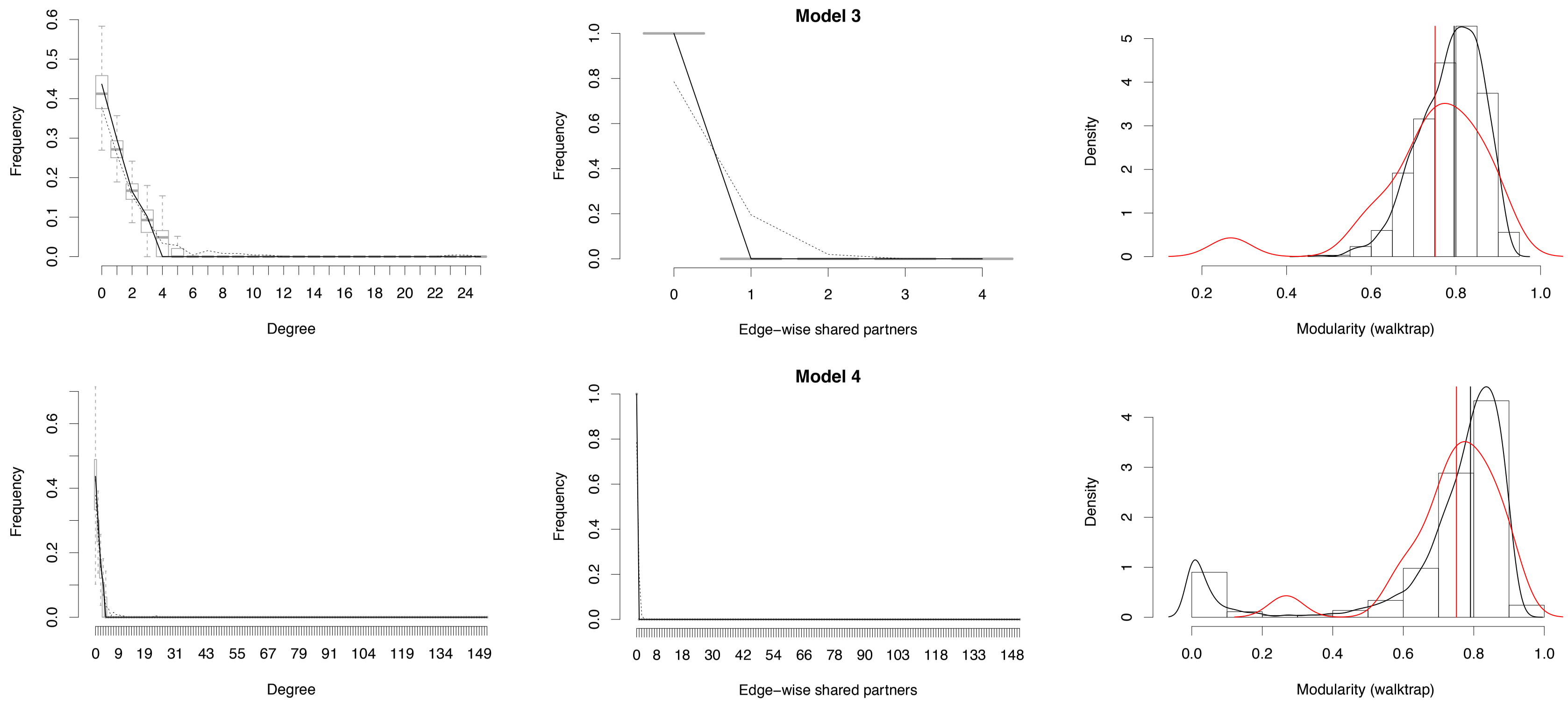

Figure S4: *In-Sample Goodness-of-Fit.* In-sample goodness-of-fit diagnostics as measured by degree, edge-wise shared partners, and modularity, for Model 3 (top row) and Model 4 (bottom row) from the main paper.
