# Supplementary material for: Multiplex communities and the emergence of international conflict
Source: PLoS One. 2019 Oct 16;14(10):e0223040. doi: 10.1371/journal.pone.0223040 (PMC6795412; doi:10.1371/journal.pone.0223040)
Supplement: S6 Fig — (PDF) [file pone.0223040.s008.pdf]

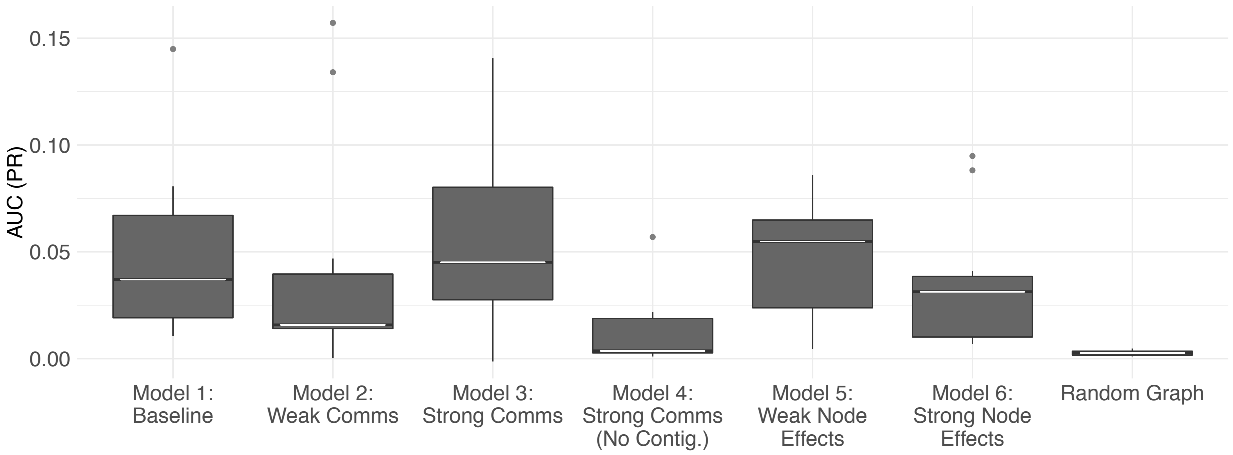

Figure S6: *Test Set Predictive Accuracy.* Out-of-sample predictive performance as area under the precision recall curve for each model in the main paper, as well as the performance of a random graph. Note that Model 5 and Model 6 omit the community bridge effect, because bridges were not present in every iteration, and thus a statistic cannot be calculated for each year.
